# THE NEXUS OF AGING AND MIGRATION: THE CURRENT STATE OF KNOWLEDGE AND WHERE GERONTOLOGICAL INQUIRIES OUGHT TO HEAD

**DOI:** 10.1093/geroni/igad104.1632

**Published:** 2023-12-21

**Authors:** Sandra Torres, Alistair Hunter, Allen Glicksman

## Abstract

Research on older ethno-racialized minorities (which comes primarily from North America) has dominated ethno-gerontological debates over the past few decades. In this research, ethnicity and race have been primarily viewed as social positions that place older people at risk of social exclusion. Migrancy, however, has yet to receive adequate attention from social gerontologists, despite the centrality of migration and aging for demographic change globally in recent decades. When migrancy is considered by gerontologists, it is not uncommon for us to think of the vulnerability trope. The highly-charged political debates in which the social exclusion and deservingness of migrant populations have been at stake have undoubtedly contributed to this state of affairs. From the field of migration studies, some have argued that the nexus of aging and migration offers new lenses from which the intersections of ethnicity, race, migrancy and old age can be approached. This symposium brings together contributors to an international network that has been working to survey the current state of knowledge at the aging-migration nexus and where future research ought to head. This symposium will argue that the study of older migrants has far-reaching implications for the economy, public policy, societal cohesion and individual wellbeing, and that – as such – this is a theoretically-profuse angle of investigation for social gerontology.

